# Impact of Accessibility to Cities at Multiple Administrative Levels on Soil Conservation: A Case Study of Hunan Province

**DOI:** 10.3390/ijerph191811768

**Published:** 2022-09-18

**Authors:** Yunzhe Dai, Xiangmei Li, Dan Wang, Yayun Wang

**Affiliations:** 1 Collaborative Innovation Center for Emissions Trading System Co-Constructed by the Province and Ministry, Wuhan 430205, China; 2 School of Low Carbon Economics, Hubei University of Economics, Wuhan 430205, China; 3 Hubei Institute of Geosciences (Hubei Selenium Industrial Research Institute), Wuhan 430034, China

**Keywords:** soil conservation, accessibility, multiple administrative levels, Hunan province

## Abstract

The development of traffic infrastructure involves massive land use changes along the transportation routes and stimulates urban sprawl at transfer nodes, leading to a degradation in ecosystem services, including soil conservation. For developing countries, especially for China, it is very important to differentiate the influences between different standards of traffic infrastructure associated with the different administrative levels of the regions where they are constructed on soil conservation. In this study, we attempt to analyze the differences in the influence of accessibility at different levels on soil conservation, for the case study area in Hunan province in China. The results indicate that: (1) traffic conditions in Hunan province have witnessed continuous improvement, and the time taken to access mega-cities, prefecture-level cities, and county-level cities from various regions has been significantly reduced. (2) The total annual soil conservation in Hunan province is maintained at approximately 2.93 × 10^9^ t. However, the spatial heterogeneity shows severe degradation in regions with lower accessibility, and weak enhancement in regions with higher accessibility. (3) A negative spatial autocorrelationship exists between accessibility and soil conservation at all levels, with the increase of administrative rank of the destination making it more obvious and intense, along with an increased tendency for the spatial distribution to concentrate. (4) Building more railways and highways from prefecture-level cities with LH clusters nearby as transfer nodes, instead of the construction of national roads and provincial roads that diverge from these railways and highways, will help limit the massive expansion of construction land and soil erosion within prefecture-level cities, rather than spreading to towns of LH clusters. This research provides an important scientific basis for future regional planning and traffic infrastructure construction, and also a reference for traffic infrastructure development in other geographically similar regions on a synchronous development stage in the world.

## 1. Introduction

In developing countries, traffic infrastructure is closely related to social and economic development [1] and residents’ welfare [2,3,4]. It even directly determines the direction and scale of urban expansion [5]. However, the sustainability of regional development becomes fragile due to such problems as food security (loss of cultivated land) [6], biodiversity reduction (loss of ecological land) [7], traffic congestion [8], increased energy consumption [9], and intensified pollutant emission [10,11] brought about by urban expansion. Since the reforms in China and the opening up of the country starting in 1978, the rapid construction of China’s traffic network has greatly optimized the circumstances for the development and utilization of its territory, strongly supporting the rapid and steady growth of China’s economy, and speeding up the process of urbanization over the past 30 years [12]. However, excessively rapid economic growth and urban sprawl will inevitably lead to the extensive utilization of natural resources, and result in more environmental issues, such as soil erosion [13], reduction in biodiversity [14], as well as degradation of air [15] and water quality [16]. These issues seriously threaten the sustainability of the socio-ecological system [17]. The proper resolution of environmental issues has become a crucial agenda in China’s national development strategy. Moreover, assessing the environmental impact of traffic infrastructure construction is an indispensable part of protecting and improving the ecological environment.

Ecosystem services have a direct bearing on human survival and development, and is functioning as important interfaces between humans and the ecological environment [18]. The construction of traffic infrastructure requires a large land area, which has led to further encroachment of agricultural land and the natural ecosystem along the traffic lines [19]. Related studies have shown that during the last 30 years, the built-up area has expanded to nearly three times in the process of rapid urbanization in China, which indicates the fragmentation of land use from a landscape ecological perspective [20]. Among the various activities involved in this process, the construction of traffic infrastructure has been the main influencing factor that disturbs the eco-environment at an unprecedented speed, resulting in the significant degradation of land ecosystem services [21]. As such, how to reduce the negative impact of land use changes on ecosystem services and ensure regional ecological security has become the focus of researchers all over the world [22,23,24].

Soil conservation is very significant to soil formation and plant root fixation. It can effectively reduce environmental damage caused by soil erosion, and also ensure food production conditions. Parent material, vegetation cover, precipitation, topography and geomorphology are collectively determining the capability of soil conservation. Parent materials dominate soil physical and chemical properties, which determine soil erosion resistance [25]; it is stable on a macro scale, but can be artificially altered on a micro scale, which leads to a significant negative effect by agricultural production [26]. Vegetation is able to increase soil organic matters, improve aggregate stability and reduce soil erosion [27]; it can be easily altered by human activities and changes soil conservation magnificently within a few years [28]. Precipitation is one of the kinetic energy sources of soil erosion [29]; it was originally determined by the local climate, but has been polarized by global warming in recent years [30]. The steeper the terrain, the greater the potential gravitational energy it provides for soil erosion [31]; on a micro scale, the construction of traffic infrastructures will weaken the original stability of the terrain [32], which furtherly increases soil erosion. Thus, soil conservation is highly sensitive to land use changes, and is an important factor impacting other ecosystem services such as water conservation, carbon storage, environmental purification, etc. Therefore, the relationship between soil conservation and land use changes has been a cause of wide concern, and a subject of extensive discussion [33,34].

Previous studies generally conclude that the change of vegetation cover caused by the expansion of urban and agricultural space is the most important and direct reason for the degradation of soil conservation [35]. A specific example is the conversion of woods and grassland with higher vegetation coverage, to cultivated land and built-up area, which are the main reasons for the degradation of soil [36], and that restoring cultivated land to woods and grassland would significantly improve soil conditions [37]. However, there is evidence that the construction of traffic infrastructure, such as railway [38] and expressway [39], will change the topography along the route, especially in mountainous and hilly areas. Additionally, due to the significant enhancement of traffic accessibility, human activities will aggregate beside the route (intensified at nodes of the transportation network) [40]. Intensified anthropogenic activities will eventually accelerate the expansion of urban [41] and agricultural spaces [42], which degrades the soil conservation [43]. These studies theoretically point out a mechanism. The traffic infrastructure construction assembles human activities at the transfer node and along the route, which leads to construction land expansion and ultimately causes the degradation of soil conservation regionally. Scarce quantitative analysis and verification of this mechanism has been conducted [44], which has very important implications for this study. Yet, the existing research has not considered that traffic infrastructure with different road types will significantly differentiate impacts on soil conservation. Considering the area scale of traffic infrastructure construction and the construction land expansion it stimulates, such differences cannot be ignored [45]. Once this issue is clarified, it helps in regulating and controlling the balance between traffic infrastructure construction and soil conservation. The territory of Hunan province encompasses almost all types of topography and landforms, and is an important province of agriculture and ecology in China. As a vital transition zone between the eastern coastal areas and western regions of China, Hunan province has experienced rapid development in traffic infrastructure in recent decades, accompanied by dramatic land use change and ecological degradation. Undertaking a case study of Hunan province, we evaluate the accessibility to cities at multiple administrative levels, and soil conservation of Hunan province at the township scale, to analyze the spatial correlation between the two indicators in different periods so as to reveal the temporal and spatial characteristics of interactions between traffic infrastructure construction and soil conservation. The study provides a scientific reference for planning future traffic networks and landscape patterns within the national territory, with an agenda of preserving and optimizing soil resources.

## 2. Materials and Methods

### 2.1. Study Area

**Figure 1:**
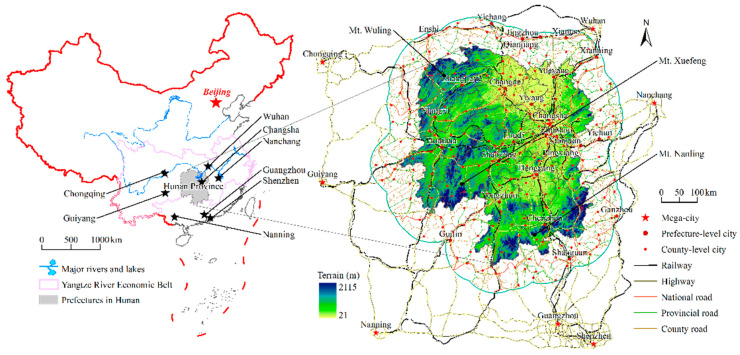
Location and traffic network of Hunan province and surrounding areas.

Hunan province is located along the mid reaches of the Yangtze River, south of Dongting Lake and north of Nanling Mountain, between 108°47′ and 114°15′ E longitudes, and 24°39′ and 30°08′ N latitudes. It borders Hubei to the north, Jiangxi to the east, Chongqing and Guizhou to the west, and Guangdong and Guangxi to the south (Figure 1). The total area of Hunan province is about 211,769 km^2^, with a total population of 69.183 million as of 2019. The territory encompasses almost all types of topography and landforms, including plains, basins, hills, mountains, rivers and lakes. It is an important province for agriculture and ecology in China, with cultivated land, woods, grasslands and swamps forming 28.69%, 61.34%, 3.15%, and 3.74% of the total area, respectively. Driven by national strategies such as the “Yangtze River Economic Belt” and “City Groups in the Middle Reaches of the Yangtze River,” Hunan province is gradually growing into a new economic hub in China. In 2020, the GDP of Hunan province reached 4178.15-billion-yuan, accounting for 8.86% of the GDP of the Yangtze River Economic Belt and 37.67% of the GDP of three provinces (Jiangxi, Hunan and Hubei) along the mid reaches of the Yangtze River, with its urbanization rate having reached 56.02%. It is worth noting that the proportion of built-up area has increased rapidly from 1.16% (2493.44 km^2^) in 1995 to 2.13% (4549.31 km^2^) in 2015.

Hunan province plays a role in connecting traffic along the east–west and north–south directions in the national road network of China. There are expressways and railways in all directions that impact the ecological environment. Railways and highways modify the original landscape and disturb the community structure of animals and plants [17,46], which directly threatens the soil conservation of the surrounding areas [47]. As a major province with a forest coverage up to 60%, and guided by the vision “to step up conservation of the Yangtze River and stop its over-development,” propounded by Chairman Xi [48], Hunan Province bears an important responsibility for the ecological protection of the whole of China.

By 2015, the total length of railway lines in Hunan province has exceeded 4500 km, with the length of the high-speed railway network reaching 1100 km. There are 7 lines, including the Beijing–Guangzhou, Shanghai–Kunming, Xiang–Gui, Shi–Chang, Luo–Zhan, Jiao–Liu, and Yu–Huai lines, forming the “three horizontal and two verticals” railway traffic network. The total length of highways exceeds 23,600 km, of which 5493 km are expressways. Moreover, the road network density of Hunan province has reached 1.81 km/100 km^2^, leading to the ecological fragmentation of land, further intensifying the negative impacts on soil conservation. As a result, issues such as conversion of cultivated land into construction land on a large scale for urbanization needs have gradually emerged, and have started to bring about a negative impact on soil conservation [49].

### 2.2. Research Methods

#### 2.2.1. Research Framework

**Figure 2:**
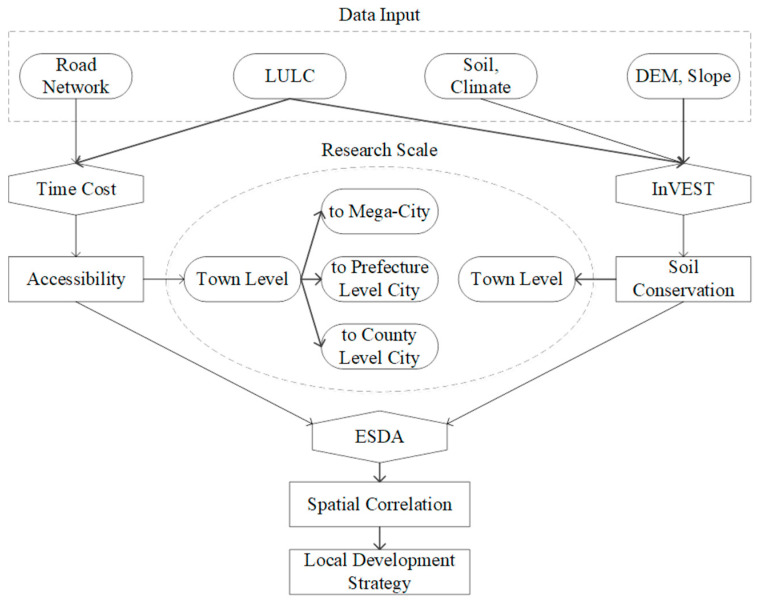
Research framework.

The research framework is composed of three steps, as shown in Figure 2. First, the accessibility from any point in the study area to the nearest county, prefecture, and mega-city is determined, according to the time impedance of different roads and land use types involved, based on China Traffic Road Network and LULC (land use/land cover) data. Next, the soil conservation of the study area is evaluated using the InVEST model, based on soil, climate, elevation, and slope data from LULC. Finally, the relationship between accessibility to cities at different administrative levels and soil conservation is analyzed, to reveal their spatial heterogeneity at the township level by using the bivariate spatial autocorrelation analysis in GeoDa, an ESDA (Exploratory Spatial Data Analysis) software (Version: 1.20, Center for Spatial Data Science, Chicago, IL, USA.).

#### 2.2.2. Measurement of Accessibility

The time taken to access a particular destination from any point in a given area reflects the convenience of spatial connection [50]. In this article, we use the average minimum impedance from the center point of the evaluation unit to all destination points as the indicator of regional traffic conditions. This is achieved by applying the Time Cost method in network analysis, which uses the minimum impedance-based accessibility analysis [51]. Time cost is measured as the average time (in minutes) required to travel 1 km, and is given as follows.

(1) $$cost=\frac{1}{v}\times 60$$

where cost is the time cost, and v is the operating speed of different modes of traffic, and is assigned to the time cost of each vector layer based on the set speed from Table 1; the vector file is then rasterized. As such, the value of the raster pixel is known as the time cost value. The distribution map of time cost can be obtained by spatially superimposing the time cost of various grades of roads.

The time cost method is used to calculate the accessible time from anywhere (a random pixel in research area) to a mega-city/prefecture-level city/county-level city nearby, which can fully reflect the spatial topological relationship of network nodes, and is widely used in traffic geography [52]. In this work, a mega-city must meet at least one of the requirements below: (1) it has a population over 10 million, (2) or it is a provincial capital; a prefecture-level city is an administrative division under the jurisdiction of provinces; a county-level city is under the jurisdiction of the prefecture-level city, and can be ultimately subdivided into several towns (the lowest administrative unit and transitional zone between urban and rural areas). The formula is as follows.

(2) $$T_{i}=\frac{1}{n-1}\overset{n}{\underset{j=1}{\sum }}d_{ij}$$

where $T_{i}$ represents the average time taken to access (hereafter referred to as access time) node i from all units (every single pixel) in the traffic network that are being evaluated. The smaller the value of $T_{i}$, the shorter the average access time to node i and better the accessibility, and vice versa. *d_ij_* represents the lowest impedance between nodes i and j. The time cost is used to generate a 30 m × 30 m precision time cost raster file, and the distance corresponding to the shortest time cost in the traffic network is identified as the minimum impedance. Specifically, the calculation of time cost is carried out referring to the “Technical Standards of Highway Engineering (JTGB01-2014),” and considering the actual situation of the studied area (Table 1). When calculating accessibility to county-level cities and prefecture-level cities, an 80 km buffer zone outside Hunan province referred to as the 1 h maximum outer range of national road is considered to supplement the original study area.

#### 2.2.3. Measurement of Soil Conservation

This study adopts the SDR (sediment delivery ratio) module in the InVEST model, which is more accurate and realistic than the traditional, general soil loss equation. Additionally, the model includes different vegetation types of the sediment retention. The specific formulae are as follows.

(3) $$SEDRET_{x}=R_{x}\cdot K_{x}\cdot LS_{x}\cdot \left(1-C_{x}\cdot P_{x}\right)+SEDR_{x}$$

(4) $$SEDR_{x}=SE_{x}\overset{x-1}{\underset{y=1}{\sum }}USLE_{y}\overset{x-1}{\underset{z=y+1}{\prod }}\left(1-SE_{z}\right)$$

(5) $$USLE_{x}=R_{x}\cdot K_{x}\cdot LS_{x}\cdot C_{x}\cdot P_{x}$$

where $SEDRET_{x}$ and $SEDR_{x}$ are the soil retention and sediment retention of grid x, respectively, $USLE_{x}$ and $USLE_{y}$ are the actual erosion in grid x and upslope grid y, $R_{x}$, $K_{x}$, $LS_{x}$, $C_{x}$ and $P_{x}$ are the rainfall erosivity factor, soil erodibility factor, terrain factor, cover management factor and soil conservation measure factor of grid x, and $SE_{x}$ is the sediment retention efficiency of grid x.

The precipitation erosivity factor R reflects the potential soil erosion due to rainfall, and is an important factor in soil erosion analysis. The equation below uses Zhou Fujian’s calculation method [53].

(6) $$R=\left[\overset{12}{\underset{i=1}{\sum }}\left(-1.5527+0.1792P_{i}\right)\right]\times 17.02$$

where R is the average annual rainfall erosivity (MJ·mm/ha·h·a), and $P_{i}$ is the average monthly rainfall (mm).

Soil erodibility factor K is the sensitivity of soil to the erosion and transportation of the eroded media, such as through raindrop splash or surface runoff. It is a comprehensive manifestation of soil erosion resistance, and includes the effects of rainfall, runoff and infiltration. There are 14 soil types in Hunan province [54]; so far, Chinese researchers have studied the soil erodibility factor in various regions including the hilly and mountainous areas south of the Yangtze River [55], Fujian province [56,57], and Hebei province [58]. The erodibility factor K of each soil type in Hunan province is referred from the literature above.

The terrain factor LS is an important parameter for analyzing soil conservation. LS reveals the influence of topographic features on soil erosion, it represents the distance that raindrops or sediments move until their kinetic energy is completely dissipated, and could be standardized as the ratio of the standard erosion plot (slope grade of 9% at a length of 22.13 m).

The model can automatically calculate the value of LS for different slopes. Based on previous research, this study set the slope threshold to 25% [59]. When the slope is more moderate than the threshold value, LS may be calculated as follows.

(7) $$LS={\left(\frac{F_{a}\times C_{s}}{22.13}\right)}^{n}\left[{\left(\frac{\sin\left(s-0.01745\right)}{0.09}\right)}^{1.4}\right]\times 1.6$$

(8) $$n=\{\begin{array}{c} 0.5,\;S\geq 5\%\\ 0.4,\;3.5\%\leq S\leq 5\%\\ 0.3,\;1\%\leq S\leq 3.5\%\\ 0.2,\;S\leq 1\% \end{array}$$

When the slope is steeper than the threshold, LS is determined by Equation (9).

(9) $$LS=0.08\lambda^{0.35}P_{s}^{0.6}$$

(10) $$n=\{\begin{array}{c} C_{s},\;\mathrm{flow}\;\mathrm{direction}=1,\;4,\;16,\;64\\ 1.4C_{s},\;\mathrm{other}\;\mathrm{flow}\;\mathrm{directions} \end{array}$$

where LS is the terrain factor; $F_{a}$ and $C_{s}$ are the catchment accumulation threshold and size of the grid, respectively, S and $P_{s}$ are the slope in degree (°) and the slope in percentage (%), respectively, and n is the slope length index which refers the standard erosion plot.

Sediment retention efficiency (SE) reflects the process of sedimentation due to filtration by vegetation and interception during the transportation of eroded material; the change in biomass can represent the retention capacity [60]. The sediment retention efficiency of different vegetation types is input to the InVEST model database in this study [61,62].

The vegetation coverage factor C is the ratio of the amount of soil erosion under a specific vegetation cover and management status, to the amount of soil erosion in land that is cleared and left continuously fallow, representing the impact of vegetation or crops and management measures on soil loss, with a value between [0, 1]. The vegetation coverage factor (f) is calculated based on the vegetation cover as follows.

(11) $$C=\{\begin{array}{c} 1,\;f=0\\ 0.6508-0.3436\mathrm{lg}f\\ 0,\;f\geq 75\% \end{array},\;0\leq f\leq 75\%$$

Management measure factor P is the ratio of soil loss after the adoption of particular measures to normal planting along the slope within the range [0, 1] [63]. The vegetation coverage factor (usle_c) is calculated using the vegetation cover using Equation (11); the management measure factor (usle_p) is extracted from the corresponding literature [64]. The sand retention rate (sedret_eff) is based on the model database, and modified based on research from literature on similar geographical areas (Table 2) [65].

#### 2.2.4. Bivariate Spatial Autocorrelation Analysis

This study applies ESDA (Exploratory Spatial Data Analysis) module in GeoDa 1.4.6 to calculate the global Moran’s I and local Moran’s I values for the global and regional spatial autocorrelationship between accessibility to cities at different administrative levels and soil conservation in Hunan province at the township level; the corresponding LISA clusters map is shown, where towns with significant characteristics can be identified. The formula for calculating the Moran’s I of the bivariate spatial autocorrelationship is as follows [66].

(12) $$Bivariate\;Moran^{’}s\;I=\frac{x_{k}^{i}-{\bar{x}}_{k}}{\sigma_{k}}\overset{n}{\underset{j=1}{\sum }}w_{ij}\frac{x_{l}^{j}-{\bar{x}}_{l}}{\sigma_{l}}$$

where $w_{ij}$ is the weight matrix of the weight space, $x_{k}^{i}$ is the value of the attribute k of the empty cell i, $x_{l}^{j}$ is the value of the attribute l of the empty cell j, ${\bar{x}}_{k}$ and ${\bar{x}}_{l}$ are the indicators of the attributes k and l, respectively, and $\sigma_{k}$ and $\sigma_{l}$ are the variance of attributes k and l, respectively.

### 2.3. Data Sources and Data Processing

The land use/land cover (LULC) data for 1995, 2005, and 2015 produced by the Chinese Academy of Sciences Resource and Environmental Science Data Center are adopted in this study. The interpretation data divide the territory of China into six primary land types and 25 secondary land types, with a raster resolution of 30 m × 30 m. This LULC data set uses Landsat TM/ETM/OLI images as the main data source, and is produced by human–computer interaction and interpretation, the average interpretation accuracy being more than 85% [67]. The territory of Hunan Province includes 6 categories, and can be subdivided into 18 subtypes (Table 2). Major road network data including expressways, national roads, provincial roads, county roads, and main roads in the towns for 1995, 2005, and 2015 are vectorized using traffic maps of China for homologous periods after being georeferenced. The spatial distribution data on soil types are compiled from the results of the second national soil survey of China. The precipitation data are extracted from the multi-year average precipitation data available in the “1995–2015 Hunan Water Resources Bulletin,” and the Kriging method is used to interpolate the values to determine the spatial distribution of precipitation in Hunan province. The elevation data are extracted from the DEM digital elevation database produced by the geospatial data cloud platform (http://www.gscloud.cn, accessed on 18 January 2022); these values—with a raster resolution of 90 m × 90 m—are used to determine the slope in ArcGIS 10.6. All the data sources are shown as Table 3.

## 3. Results and Analysis

### 3.1. Evolution of Accessibility at Different Levels

**Figure 3:**
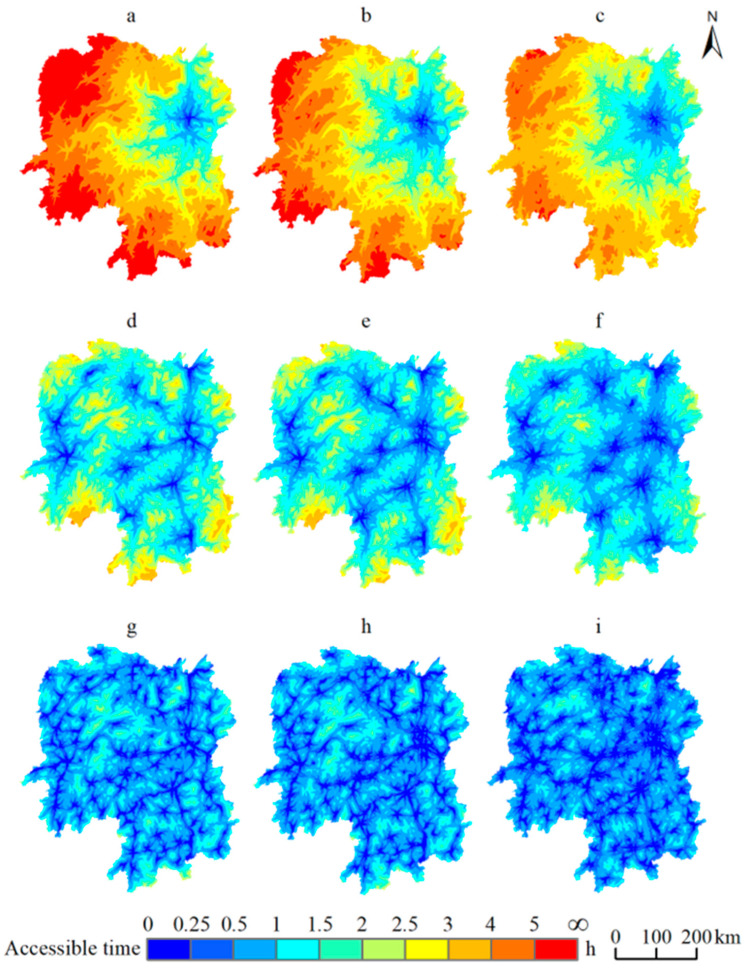
Traffic accessible time on multiple administrative levels in Hunan province: (**a**–**c**) are accessibility to mega-cities in 1995, 2005, and 2015; (**d**–**f**) are accessibility to prefecture-level cities in 1995, 2005; and 2015, and (**g**–**i**) are accessibility to county-level cities in 1995, 2005, and 2015.

From Figure 3a–c, it can be seen that during 1995–2015, the average access time to mega-cities decreased from 3.21 h to 2.51 h, and for the town with the highest time cost, it decreases from 6.86 h to 5.05 h. For 1995—considering Changsha City as the core—a 1 h traffic circle radiating outward is formed. There are vast areas in Wuling Mountain, Xuefeng Mountain and Nanling Mountain that require more than 6 h to access a mega-city. The Dongting Lake watershed and the Mid-Hunan basin are at short distances to mega-cities such as Wuhan, Guangzhou, and Shenzhen, and access times to mega-cities are generally within 3 h. By 2005, the 1 h traffic circle has expanded significantly, and areas with access times above 5 h had shrunk into a small range, covering only steep mountains with extremely low population densities. In 2015, the 1 h traffic circle covers the Changsha–Zhuzhou–Xiangtan (CZT) urban agglomeration, and the access time for large cities in Wuling Mountain drops to approximately 4 h, and the access time for Nanling Mountain and Xuefeng Mountain drops to around 3 h. This shows that the ease of communicating with the outside world has greatly improved over the years, in faraway mountainous areas.

It can be seen from Figure 3d–f that the average access time to prefecture-level cities decreases from 1.20 h to 0.91 h. For the town with the highest time cost, it decreases from 3.26 h to 2.54 h from 1995 to 2015. The access time to prefecture-level cities is reduced by the 1 h traffic corridor serving multiple areas with low accessibilities to prefecture-level cities. In 1995, the continuous wide strip including the northern foothills of the Wuling Mountain, the southern foothills of the Luoxiao Mountain, the Xuefeng Mountain, and the Nanling Mountain needs more than 3 h to access a prefecture-level city. The 1 h traffic corridor has clearly widened by 2005, but the traffic conditions in the far west have not seen a significant improvement. By 2015, the 1 h traffic corridor grows from a linear path into a network, forming a contiguous area, with an access time of less than 1 h for the hilly basin of Hunan. The northern foothills of the Wuling Mountain and the southern foothills of the Xuefeng Mountain remain the only scattered areas requiring longer than 3 h. Thus, the traffic conditions in the Nanling Mountain and Luoxiao Mountain have greatly improved.

Figure 3g–i demonstrate that with the provincial roads and county roads subdivided into multiple 0.5 h traffic loops with higher density, the average time of access for county-level cities decreases from 0.63 h in 1995 to 0.50 h in 2015, and for the town with the longest access time, the average access time decreases from 1.13 h to 1.04 h. In 1995, the 0.5 h traffic loops are scattered across Hunan province, and the expansive areas in the Wuling Mountain, Xuefeng Mountain, Nanling Mountain and Luoxiao Mountain need more than 1.5 h to accesses a county-level city. The situation in 2005 is roughly the same as in 1995, with the coverage from 0.5 h traffic loops not expanding significantly; only the county-level cities in the CZT urban agglomeration show a considerable reduction in time cost. By 2015, the 0.5 h traffic network has developed extraordinarily, and a considerable length of provincial and county roads has been built in various mountainous areas; the access time of towns to a county-level city drops to about 0.75 h.

### 3.2. Evolution of Soil Conservation

Soil conservation is classified into five grades, i.e., low, mid-low, mid, mid-high, and high, corresponding to 0–10 t/ha, 10–50 t/ha, 50–100 t/ha, 100–200 t/ha, and more than 200 t/ha, respectively. The study results show that the amount of soil conservation in Hunan Province is maintained at about 2.93 × 10^9^ t throughout the year. In 1995, areas with mid-high and high grade conservation accounted for 44.05% of the soil conservation, and the average amount of soil conserved was 138.93 t/ha.

By 2005, the areas with high and mid-high grade conservation decrease to 43.96%, and the average amount of soil conserved decreases slightly to 138.47 t/ha. This is mainly due to a decrease in the area with high grade conservation from 29.43% to 29.35%, and an increase in the area with low grade conservation from 24.92% to 25.01%. Moreover, the changes in the spatial distribution of soil conservation in the Wuling Mountain and Dongting Lake basin change drastically during this period. The areas with increases and decreases of soil conservation in the Wuling Mountain and Nanling Mountain are relatively concentrated in high-altitude mountainous areas, contributing simultaneously to desertification control and cultivated-land expansion. Meanwhile, the vegetation height and canopy density of newly planted woods have not yet fully developed, and soil conservation here is insufficient. The soil conservation in the Dongting Lake basin has declined significantly, and swamps beside the water bodies being converted to cultivated land have become a common phenomenon. With new railways and motorways becoming functional, the built-up area in the CZT urban agglomeration continues to expand rapidly. As the built-up area is more resistant to soil erosion than the cultivated land, the level of soil conservation increases greatly. It should be noted that the expansion of construction land is mainly proceeded by occupying cultivated land. However, under a strict farmland protection regime in China (cultivated land protection is given higher priority than ecological land), the loss of ecological land will be further caused. Considering that, covering cultivated land with construction land will locally enhances soil conservation, but globally, it will lead to more serious soil erosion.

**Figure 4:**
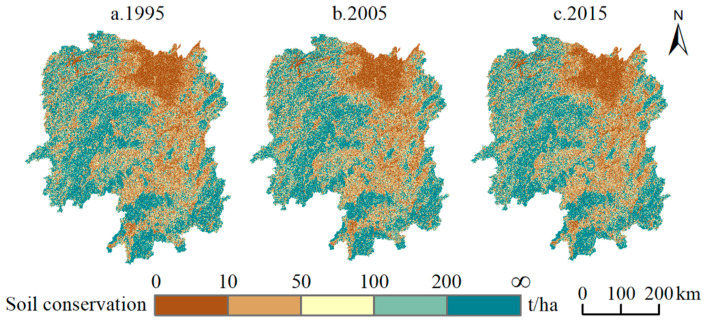
Spatial distribution of soil conservation in Hunan province, 1995–2015.

By 2015, the areas with high and mid-high grade conservation increase to 44.17%, and the high-grade areas increase to 29.55%, with the average amount of soil restored being 138.77 t/ha (Figure 4). With respect to the spatial distribution of changes in soil conservation, the contribution from the conversion of swamps in the upper reaches of the Dongting Lake basin into cultivated lands is tremendous, as is the conversion of a large number of water bodies in the downstream areas to swamps. The degradation of soil conservation results in a large area with high grade soil conservation being transformed into a mid-grade area. In Wuling, Xuefeng and the low-altitude foothills of the Nanling Mountain, large scale wood land has been converted to cultivated land, and soil conservation has degraded. The spread of soil degradation to surrounding towns has happened simultaneously with the development of traffic infrastructure.

### 3.3. Influence of Accessibility on Soil Conservation

Throughout Hunan province, the spatial autocorrelationship between accessibility and soil conservation for 1995, 2005, and 2015 passes the significance test, and they indicate an obvious negative spatial autocorrelationship. Correlatively, villages and towns with higher accessibility generally have lower soil conservation.

From Table 4, it can be seen that the negative global spatial autocorrelationship between accessibility to mega-cities and soil conservation first strengthens and then weakens during 1995–2015. Due to the construction of traffic infrastructure—especially railways and highways mainly concentrated in the Dongting Lake area and the CZT urban agglomeration—the spatial aggregation of negative influences on soil conservation has strengthened, resulting in an increase in the absolute value of Global Moran’s I. As the traffic infrastructure develops in a diffused manner towards the west and south during 2005–2015, the absolute value of Global Moran’s I drops slightly. The spatial aggregation of the negative influences on soil conservation is weakened, and the gap between different regions is reduced.

The negative spatial autocorrelationship between accessibility to prefecture-level cities and soil conservation is similar to that at the mega-city level. However, the absolute value of Global Moran’s I is significantly smaller. Highways along routes competing gradually with railways [68] lead to lesser land exploitation, and increased accessibility to prefecture-level cities may have lesser impacts on soil conservation. With the lower construction standards of motorways at the county level, a significant reduction in land exploitation may be achieved. The autocorrelationship between accessibility to county-level cities and soil conservation is thus even weaker. As highways do not pass through every county-level city before 2005, accessibility at this level is dominated by national roads, provincial roads and county roads, and the coverage of these three levels in Hunan province is evenly distributed, resulting in the Global Moran’s I remaining almost unchanged. After 2015, highways gradually start playing a greater role in providing access to county-level cities, leading to an increase in the negative spatial autocorrelationship between accessibility to county-level cities and soil conservation.

**Figure 5:**
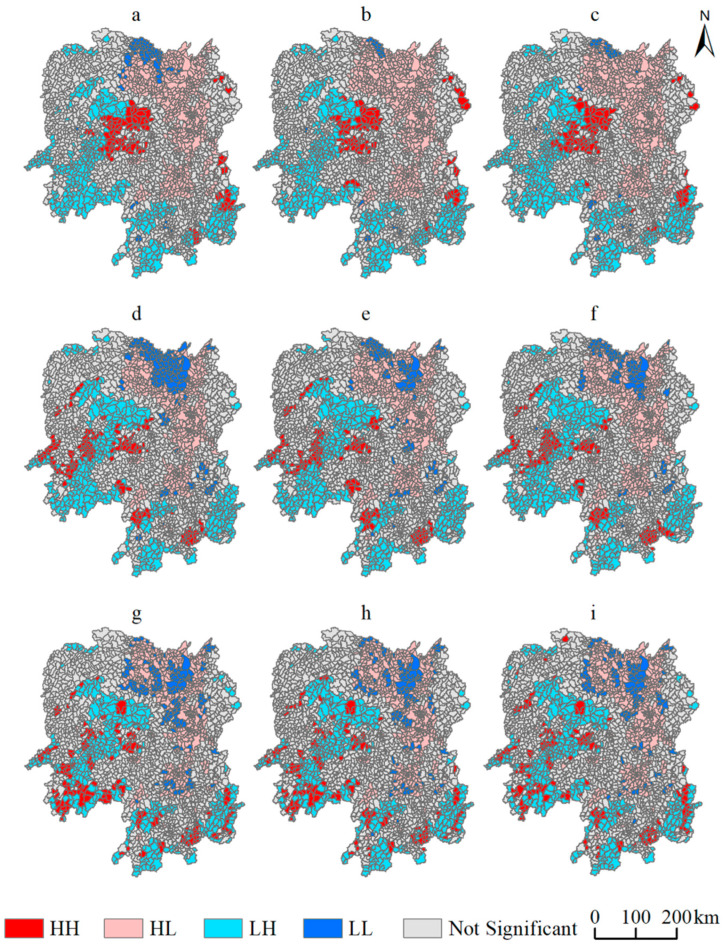
LISA cluster maps of bivariate spatial autocorrelationship between traffic accessibility to cities at different administrative levels and soil conservation from 1995 to 2015: (**a**–**c**) are the spatial autocorrelationship between traffic accessibility to mega-cities and soil conservation in 1995, 2005, and 2015; (**d**–**f**) are between traffic accessibility to prefecture-level cities and soil conservation in 1995, 2005; and 2015, and (**g**–**i**) are between traffic accessibility to county-level cities and soil conservation in 1995, 2005, and 2015.

Erosion in regions with severe undulations in terrain are more likely to impact soil conservation [31,69]. The autocorrelationship between accessibility and soil conservation could therefore present a significant spatial heterogeneity, and LISA cluster maps are helpful in understanding their specific characteristics. HH (High-High) and LL (Low-Low) clusters show a positive local spatial autocorrelationship, while the correlations of HL (High-Low) and LH (Low-High) clusters are negative. An HH cluster indicates higher accessibility of the town compared to surrounding towns, and stronger soil conservation. An HL correlation means that accessibility is higher than that of surrounding towns, but soil conservation is weaker. An LH cluster reveals low accessibility and strong soil conservation, whereas in an LL cluster pop up, accessibility and soil conservation are both in a worse condition than in surrounding towns. Figure 5 below shows the LISA cluster maps of bivariate spatial autocorrelationship between accessibility to cities at different administrative levels and soil conservation, revealing the changes from 1995 to 2015 as shown as below.

A small number of towns in HH clusters, with accessibility to mega-cities and strong soil conservation, are located at the edge of the Wuling Mountain. LL clusters are also relatively rare and concentrated in the upper reaches of Dongting Lake. HL clusters are widely distributed in the CZT urban agglomeration and the lower-middle reaches of Dongting Lake. LH clusters cover a large number of townships in the Wuling Mountain and Nanling Mountain areas. HH clusters appear when Changsha is considered as the core, and railways and highways extending radially to other parts of Hunan province dominate the accessibility to mega-cities. However, the foothills of the Wuling mountainous area not only have higher accessibility than high mountainous areas, but also maintains stronger soil conservation. The upper reaches of Dongting Lake have lower accessibility and weaker soil conservation than the lower-middle reaches, due to the steeper terrain, therefore indicating LL-related spatial autocorrelationship. The CZT urban agglomeration and the lower-middle reaches of Dongting Lake have high accessibility and weak soil conservation involving intensive land exploitation, as towns in these two areas are HL-related. The land on Wuling Mountain and Nanling Mountain is largely undeveloped, and their access times to mega-cites are high. However, due to the flourishing vegetation cover, the spatial autocorrelationship is LH-related. It is worth noticing that the number of HL clusters gradually increased during the period 1995–2015, and spread outward along highways and railways from the center of the CZT urban agglomeration.

There are large numbers of HH clusters, considering accessibility to prefecture-level cities and soil conservation, which are scattered on Wuling Mountain and Nanling Mountain with multiple roads passing through. LL clusters have also increased, which spread from the upper reaches of Dongting Lake to the middle and lower reaches. HL-related towns are widely distributed in the CZT urban agglomeration and the lower-middle reaches of Dongting Lake, while LH clusters cover most of the Wuling Mountain and Nanling Mountain regions. The main reason for the increase in these clusters is that the highways and national highways that determine accessibility to prefecture-level cities are both extending radially outwards, considering Changsha as the core. Moreover, accessibility to prefecture-level cities is obviously higher with less LL clusters than in the Dongting Lake basin, resulting in a thorough traffic network in the CZT urban agglomeration. Towns under subdivisions of the prefecture-level central districts in the Wuling, Xuefeng and Nanling mountainous areas, with relatively flat terrain undulations, have higher accessibility. The soil conservation in these areas is as strong as in surrounding towns. HH clusters have significantly aggregated, with HL and LH clusters showing increasing trends during 1995–2015.

The spatial autocorrelationship between accessibility to county-level cites and soil conservation is in synchronization with that for accessibility to prefecture-level cities, revealing aggregation of HH, LL and LH-related towns. However, HH clusters are obviously more dispersed. Intensive development of traffic infrastructure in the CZT urban agglomeration increases the allocation of cultivated land for construction purposes. With the emergence of some LL clusters in the south–central hilly basin, it can be seen that vast areas of cultivated land are allotted for construction, away from the CZT urban agglomeration. HL clusters are aggregated in the CZT urban agglomeration, and only Yueyang has enhanced accessibility, being closely connected with some cities in Hubei province. For the period 2005–2015, the construction of provincial and county roads is seen to be relatively uniform across various regions of Hunan province, as opposed to the situation of railway and highway construction, which are mainly laid out in the east of the province. Therefore, the inter-annual changes in autocorrelationship between accessibility to county-level cities and soil conservation is relatively insignificant. Lisa cluster map at different levels shows that towns with similar spatial autocorrelationships between traffic accessibility to cities at different administrative levels and soil conservation are more aggregated when taking cities with higher administrative levels as the destination, but more dispersed at lower levels. It reveals that a particular length of motorway with a higher construction standard can drive more intensive construction land expansions and soil erosion along it, which is also confirmed by works in Table 4.

## 4. Discussion

### 4.1. Objectives and Significances of This Study

How to strike a balance between traffic infrastructure construction and ecosystem services is an important issue for developing countries and regions to achieve sustainable development, especially those that develop rapidly. Being closely related to food and ecological security, soil conservation is a magnificent part of ecosystem services. Starting from the spatial autocorrelation between transportation accessibility at different levels and soil conservation, this study explores the balanced spatial optimal solution between traffic infrastructure construction and soil conservation, so as to provide a reference for future transportation planning in similar regions. Pre-existing studies have pointed out and quantitively verified that traffic infrastructure construction aggregates construction land expansion and retrogresses soil conservation [35,36,37,38,39,40,41,42,43,44]. This paper further considers that different traffic accessibilities to cities at multiple administrative levels and traffic infrastructure construction with different road types have significantly different impacts on soil conservation, and successfully explores a feasible spatial configuration solution to balance traffic infrastructure construction and soil conservation.

Multiple methods are adopted in order to achieve research objectives. In the study, the time cost method is used to calculate the accessibility to cities at multiple administrative levels. The SDR (sediment delivery ratio) module in the InVEST model is used to calculate the soil conservation. Additionally, the ESDA (Exploratory Spatial Data Analysis) module in GeoDa is finally applied for revealing the spatial autocorrelationship between the above two. The output results perspectively present the negative global spatial autocorrelationship between accessibility to cities at multiple administrative levels and soil conservation, and explicitly identify the spatial distribution. Proceeding traffic infrastructure construction in towns with a significant positive spatial autocorrelationship between traffic accessibility and soil conservation is suggested.

### 4.2. Influencing Mechanism of Accessibility on Soil Conservation

Improvements in transportation have resulted in accelerated economic growth, and disturbed cultivated land and the natural environment (woods, grassland, water bodies, and swamps). Due to China’s rigorous protection system for cultivated land, the natural environment that provides ecological services including soil conservation is considered as ultimate option for infrastructure development, leading to its destruction. The trend is for development to keep spilling over from the outskirts of the town to the suburbs. Since the proposal for converting farmland to forest in Hunan province was launched in 2000, there has been a return of cultivated land to natural habitat on a considerable scale in the Wuling and Nanling mountainous areas, and upper reaches of the Dongting Lake basin, thus balancing the conversion of ecological land to cultivated land. However, there is a certain period of time required for a tree sapling to mature and achieve full functionality with regard to soil conservation, leading to a delay in the response to land use conversion. This study validates and further explores the research framework of “Traffic Infrastructure Construction-Land Use Change-Soil Conservation Response” [70,71,72]. Based on the results of spatial autocorrelation analysis, it can be inferred that, in HL and LH clusters (towns with a significant negative spatial autocorrelationship between traffic accessibility and soil conservation), proceeding traffic infrastructure construction may lead to more serious soil erosion, which should be additionally cautious. However, in HH and LL clusters, moderate traffic infrastructure can improve accessibility with only minimal soil erosion. The above strategy suggestions can also be implemented in other regions that share similar geographical and developmental characteristics.

### 4.3. Effect of Construction of Traffic Infrastructure with Lower Standards on Soil Conservation

**Figure 6:**
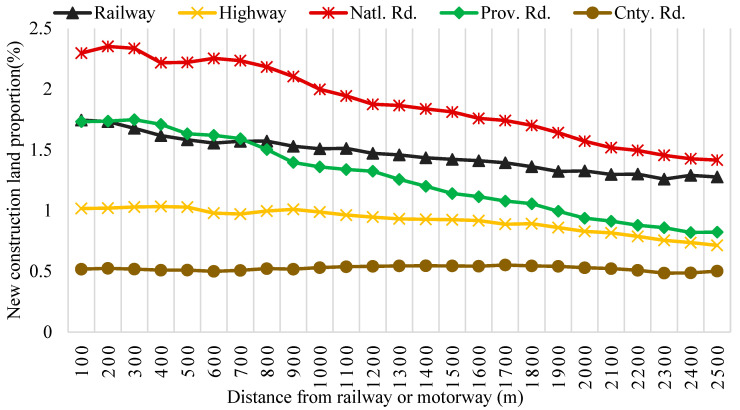
The sprawl of construction land on the road side of railways and motorways from 1995 to 2015.

It has been proven that construction of traffic infrastructure with lower standards results in longer access times to cities at multiple administrative levels, and involves less land exploitation from a macro and global perspective [73]. However, from a micro and local view, national and provincial roads bear greater responsibility for triggering construction land expansions on the side of the roads, compared to railways and highways, which impair the natural environment and soil conservation in rural areas (especially in steep mountainous areas highly sensitive to soil erosion) to a greater extent (Figure 6). It can be seen that county roads are not a crucial driver of construction land expansions (new construction land proportion is steady at all distance range form it). That is because a county road is built for transportation between a town and a county-level city, but not between two cities. Considering a town is a transitional zone between urban and rural areas, there is no strong demand for construction land expansions. Comparing with the results in Section 3.3, the global aggravation of soil erosion caused by the construction of railways and highways is actually caused by the shift of the demand for construction land expansion from the areas with higher traffic accessibility to the areas with lower traffic accessibility. Apparently, transfer to LH clusters results in more soil erosion. Overall, the construction of national and provincial roads strongly boosts construction land expansions, and should be prudently proceeded in towns with low traffic accessibility and high soil conservation (LH clusters in Figure 5).

In order to balance traffic accessibility and soil conservation in a region, an appropriate strategy should be to build more railways and highways from prefecture-level cities with LH clusters nearby as transfer nodes, while reducing the construction of national roads and provincial roads that diverge from these railways and highways. Thus, a compromise will be ultimately achieved. That is to say, the massive expansion of construction land and soil erosion will be limited within prefecture-level cities, rather than spreading to towns of LH clusters.

### 4.4. Directions for Future Research

The reasons leading to changes in soil conservation are diverse and complex, including both environmental factors (climate change, natural disasters, etc.), and socio-economic factors (agricultural production, pollutant discharge, etc.). Natural disasters such as fires [74] and storm surges [75] can cause large-scale damage to woods within a short period, leading to rapid degradation of soil conservation. In addition, overgrazing may cause grassland degradation, simultaneously impacting soil conservation [76]. In addition, wastewater discharged by highly polluting enterprises causes deterioration of water quality, and affects the growth of plants in the area where this water is supplied [77], thereby weakening soil conservation. However, this article only discusses the impact on soil conservation of land use conversion caused by infrastructure development to increase the accessibility of towns at various administrative levels. Wider environmental and socio-economic factors are not considered in sufficient detail. Exploring the driving forces of soil conservation will be the research direction in the future. In addition, due to limitations in data acquisition, the study determines accessibility based on land transportation, without considering other parameters such as those related to waterway shipping and aviation. Though the accuracy of the result is converged into an acceptable range, there is certain disagreement between the calculated and actual access times. The further research is needed to pay close attention to more accurate calculations.

## 5. Conclusions

Using the time cost method and InVEST model, this study evaluates accessibility to cities at multiple administrative levels, as well as soil conservation of Hunan province, in China, for 1995–2015, under the “Traffic infrastructure Construction-Land Use Change-Soil Conservation Response” framework. The following conclusions can be drawn from the study results. (1) A continuous improvement of the traffic network in Hunan province is detected. The CZT urban agglomeration and western mountainous areas are well connected. (2) Scattered but severe degradation of soil in faraway mountainous areas (with low accessibility), and slight enhancement in soil conservation in a wide area along railways and motorways (with high accessibility) are simultaneously observed in Hunan province. (3) The negative spatial autocorrelationship between accessibility to cities and soil conservation—considering multiple administrative levels—indicates that the correlations are stronger when the administrative levels of the destinations are higher. The negative impact from high accessibility in the CZT urban agglomeration is highly pronounced at all administrative levels, and must be considered in urban and rural planning. (4) Building more railways and highways from prefecture-level cities with LH clusters nearby as transfer nodes, instead of the construction of national roads and provincial roads that diverges from these railways and highways, will help limit the massive expansion of construction land and soil erosion within prefecture-level cities, rather than spreading to towns of LH clusters. These findings provide guidance for the alleviation of the problem of degradation of soil from traffic infrastructure construction, and for enhancing the efficiency of trade-offs between urbanization and ecological preservation under a precondition of sustainable development.

## Figures and Tables

**Table 1:** Time cost of different spatial objects.

Spatial Object	Highway	National Road	Provincial Road	County Road	Railway	Land	Water
speed (km/h)	100	80	60	50	120	5	1
time cost (min/km)	0.6	0.75	1	1.2	0.5	12	60

**Table 2:** Parameters of soil conservation evaluation model.

Primary Land Types	Secondary Land Types	Lucode	usle_c	usle_p	sedret_eff
Cultivated land	Paddy	11	217	0.20	50
	Dryland	12	291	0.45	25
Woods	Woods	21	86	1.00	55
	Shrub	22	156	1.00	50
	Sparse woods	23	367	1.00	45
	Other woods	24	166	0.15	30
Grassland	High coverage grassland	31	315	1.00	40
	Moderate coverage grassland	32	130	1.00	40
	Low coverage grassland	33	373	1.00	35
Waters	River	41	0	0.00	0
	Lake	42	0	0.00	0
	Reservoir and pond	43	0	0.00	0
	Beach	46	411	1.00	5
Construction land	Urban	51	0	0.00	0
	Rural residential land	52	0	0.00	0
	Other built-up land	53	0	0.00	0
Unused land	Swamp	64	429	1.00	60
	Bare land	65	1000	1.00	0
	Bare rock gravel land	66	1000	1.00	0

**Table 3:** Basic information of input data.

Data	Source	Form	Resolution	Duration
LULC	Resource and Environmental Science Data Center of Chinese Academy of Sciences	Raster	30 m × 30 m	1995–2015
Traffic Network	Vectorized from China Traffic Maps	Vector	-	1995–2015
Soil	Rearranged from the Second National Soil Survey of China	Raster	30 m × 30 m	-
Precipitation	Multi-year averaged and Kriging interpolated from Hunan Water Resources Bulletin	Raster	30 m × 30 m	-
DEM	Geospatial Data Cloud Platform	Raster	90 m × 90 m	-
Slope	Calculated from DEM using ArcGIS 10.6	Raster	90 m × 90 m	-

Note: All data above are resampled to a 30 m × 30 m resolution.

**Table 4:** Global Moran’s I of bivariate spatial autocorrelationship between traffic accessibility to cities at different administrative levels and soil conservation from 1995 to 2015.

Years	AMC/SC	APC/SC	ACC/SC
1995	−0.513	−0.395	−0.328
2005	−0.566	−0.461	−0.337
2015	−0.557	−0.447	−0.371

Abbr: AMC/APC/ACC—accessibility to mega-cities/prefecture-level cities/county-level cites; SC—soil conservation.

## Data Availability

The original Landsat TM/ETM/OLI images for land use/land cover (LULC) data production and the DEM digital elevation database in this study can be download at http://www.gscloud.cn, (accessed on 18 January 2022) for free.
